# Draft Genome Sequence of *Salmonella enterica* subsp. *enterica* Serovar Kiambu Strain CRJJGF_00061 (Phylum *Gammaproteobacteria*)

**DOI:** 10.1128/genomeA.00588-16

**Published:** 2016-07-07

**Authors:** Sushim K. Gupta, Elizabeth A. McMillan, Charlene R. Jackson, Prerak T. Desai, Steffen Porwollik, Michael McClelland, Lari M. Hiott, Shaheen B. Humayoun, Jonathan G. Frye

**Affiliations:** a Bacterial Epidemiology and Antimicrobial Resistance Research Unit, U.S. National Poultry Research Center, U.S. Department of Agriculture, Agricultural Research Service, Athens, Georgia, USA; b Department of Microbiology, University of Georgia, Athens, Georgia, USA; c Department of Microbiology and Molecular Genetics, University of California Irvine, Irvine, California, USA

## Abstract

We report a 4.58 Mbp draft genome sequence of *Salmonella enterica* subsp. *enterica* serovar Kiambu strain CRJJGF_00061 isolated from cattle in 2004.

## GENOME ANNOUNCEMENT

The genus *Salmonella* is one of the major zoonotic food-borne pathogens and an important public health concern worldwide which can be transmitted to humans via contaminated food or water causing sporadic cases or outbreaks of salmonellosis (1, 2). Food animals and their products are associated with *Salmonella* infections. Animal contact represents another source of human infection and a threat to public health (3). There are over 2,579 *Salmonella* serovars, however, less than 100 serotypes account for most human infections (e.g., Enteritidis, Typhimurium, Newport, and Heidelberg) (4, 5).

*Salmonella* strain CRJJGF_00061 was isolated from cattle using standard microbiology methods. The isolate was serotyped using SMART (6). The isolate was serogrouped using serogroup-specific antisera (Difco Laboratories, Detroit, MI) and serovar was determined at the National Veterinary Services Laboratories, APHIS, USDA (Ames, IA). This bacterium belongs to antigenic group O:4(B), along with *Salmonella* serovar Nakuru, and contained somatic O antigens 1,4,12, and flagellar H antigens z (phase 1) and 1,5 (phase 2) (1,4,12:z:1,5) (7). A study in 2007 reported that 4% of positive multi-drug resistant (MDR) *Salmonella* isolates from Australia were *Salmonella* serovar Kiambu (8). Using pulsed-field gel electrophoresis (PFGE) as described by PulseNet (9), the isolate was assigned PFGE pattern TENX01.0005. MICs (µg/ml) were determined by broth microdilution using the Sensititre semi-automated antimicrobial susceptibility system (TREK Diagnostics Systems, Thermo Fisher Scientific, Inc., Oakwood Village, OH). Results were interpreted according to Clinical and Laboratory Standards Institute (CLSI) guidelines (10).

Genomic DNA was isolated using GenElute bacterial genomic DNA kit (Sigma-Aldrich, St. Louis, MO), the DNA library was constructed using the Nextera-XT DNA preparation kit, and paired-end sequencing was performed on the Illumina HiSeq2500 (Illumina Inc., San Diego, CA) using a 500-cycle MiSeq reagent kit. About 3,520,806 reads with quality score ≥30 were assembled using Velvet assembler (11), resulting in 168 contigs with minimum contig length ≥200 bp. The total assembly size was 4.58 Mbp, with *N*_50_ values of 66.2 kbp, and G+C content of 52.10%. The contigs were ordered with Mauve (12) using *Salmonella* LT2 as reference, and prodigal (13) and ARAGORN (14) were used to predict protein coding sequences (CDS) and tRNAs. A total of 4,277 coding sequences (≥50 amino acids) and 42 tRNAs were predicted within the genome. Prophages, signal peptides, and resistance genes were predicted using PHAST (15), signalp (16), and ARG-ANNOT (17), respectively. We identified signal peptides in 418 genes, two clustered regularly interspaced short palindromic repeat (CRISPR) (18) loci usually associated with a CRISPR-Cas system, and four phages in the genome. We detected one resistance gene, *Aac6-Iy*, which remains cryptic and located on the chromosome (19). No phenotypic antimicrobial resistance was detected. The detected arsenic resistance genes (*pst*BACS) (20) were correlated with phenotypes. The tested MIC of the isolate was 206 µg/ml for arsenate and 29 µg/ml for arsenite compared to wildtype *Salmonella* which had a median MIC of 51 µg/ml for arsenate and 15 µg/ml for arsenite. To our knowledge, no other genomic data for *Salmonella* serovar Kiambu exists in the literature. Addition of new serovars draft genomes increases the diversity of *Salmonella* genomes currently available for comparative analysis.

### Nucleotide sequence accession number.

Genome sequences of *Salmonella enterica* subsp. *enterica* serovar Kiambu strain CRJJGF_00061 have been deposited in GenBank under the accession number JQUR00000000. This paper describes the first version.
